# Nonpher: computational method for design of hard-to-synthesize structures

**DOI:** 10.1186/s13321-017-0206-2

**Published:** 2017-03-20

**Authors:** Milan Voršilák, Daniel Svozil

**Affiliations:** 1 CZ-OPENSCREEN: National Infrastructure for Chemical Biology, Laboratory of Informatics and Chemistry, Faculty of Chemical Technology, University of Chemistry and Technology Prague, Prague, Czech Republic; 2 CZ-OPENSCREEN: National Infrastructure for Chemical Biology, Institute of Molecular Genetics, AS CR v.v.i., Prague, Czech Republic

**Keywords:** Synthetic feasibility, Molecular complexity, Molecular morphing

## Abstract

In cheminformatics, machine learning methods are typically used to classify chemical compounds into distinctive classes such as active/nonactive or toxic/nontoxic. To train a classifier, a training data set must consist of examples from both positive and negative classes. While a biological activity or toxicity can be experimentally measured, another important molecular property, a synthetic feasibility, is a more abstract feature that can’t be easily assessed. In the present paper, we introduce Nonpher, a computational method for the construction of a hard-to-synthesize virtual library. Nonpher is based on a molecular morphing algorithm in which new structures are iteratively generated by simple structural changes, such as the addition or removal of an atom or a bond. In Nonpher, molecular morphing was optimized so that it yields structures not overly complex, but just right hard-to-synthesize. Nonpher results were compared with SAscore and dense region (DR), other two methods for the generation of hard-to-synthesize compounds. Random forest classifier trained on Nonpher data achieves better results than models obtained using SAscore and DR data.

**Electronic supplementary material:**

The online version of this article (doi:10.1186/s13321-017-0206-2) contains supplementary material, which is available to authorized users.

## Background

Virtual screening is a well-established approach in which possible biologically active molecules are searched in the large collections of available screening compounds [1, 2]. However, virtual screening generates structures mostly similar to known ones. If new chemotypes are to be identified, virtual compounds can be assembled from scratch using de novo design [3] that, typically, generates thousands of potentially novel compounds. Because it is unrealistic to synthesize and test so many compounds [4], their synthetic accessibility is assessed and compounds difficult to synthesize are removed from the virtual library. The assessment of compound synthetic feasibility can be done either manually, or computationally. But, due to a large number of structures, a manual examination is usually impractical. In addition, it has been demonstrated that medicinal chemists are not very consistent in identifying synthetically unfeasible compounds [5–7]. Thus, computational methods were developed as an alternative means for compound synthetic feasibility assessment [8]. These can be roughly divided into two groups [9]. In a retrosynthetic approach [10–13], a target molecule is decomposed to starting materials by breaking bonds that can be easily created by known chemical reactions. The retrosynthetic approach requires the databases of both starting materials and reactions annotated with yields and reaction conditions. Not only that keeping these databases up-to-date is a difficult task, but retrosynthetic methods are also, due to their high computational demands, not suitable for large-scale predictions. Another approach of synthetic accessibility assessment is based on the complexity of a structure itself. An assumption behind this approach is that more complex structures are harder to synthesize. However, due to the ambiguous and context dependent definition of molecular complexity [14], its evaluation is not an easy task. Simple and commonly used complexity metrics is a molecular weight. More sophisticated complexity measures (e.g., Bertz [15], Whitlock [16], BC [17], or SMCM [18] indices) are calculated from a number of atoms, bonds, rings, and/or hard-to-synthesize motifs, such as chiral centers or uncommon ring fusions. While a complexity approach is fast enough for primary screening, it has also its limitation: it removes complex molecules that can be actually synthesized from already existing complex precursors. To overcome this problem, Ertl suggested [9] an SAscore prediction model based on the occurrence of molecular circular fragments [19] in the database of synthetically accessible compounds.

Another way how to assess synthetic accessibility is to use supervised machine learning approaches [20], such as support vector machines, artificial neural networks or random forests (RF). To train a binary classifier requires a training data set consisting of both positive (i.e., easy-to-synthesize) and negative (i.e., hard-to-synthesize) examples. While positive examples can be selected from the database of existing compounds, such as PubChem [21] or ZINC [22], no equivalent database is available for negative examples. Nevertheless, as negative examples can be used compounds with SAscore higher than 6 [9]. Alternatively, in a dense region (DR) approach [20], easy-to-synthesize compounds are identified as these coming from the dense and hard-to-synthesize compounds from the sparse regions of chemical space. For a given compound, chemical space density is evaluated by calculating the number of its nearest neighbors. However, both SAscore and DR methods assess already existing structures a majority of which is, in principle, amenable to synthesis. Thus we developed Nonpher, a method for the construction of hard-to-synthesize virtual compounds which is based on a previously published molecular morphing approach [23]. Using Nonpher, a virtual library of 1,706,950 hard-to-synthesize compounds was constructed (Additional file 1). This library was then used to build a random forest classification model. The quality of this model was verified by predicting the synthetic accessibility of 40 hard-to-synthesize compounds collected carefully from literature. In addition, this model outperformed similar models trained on data constructed by SAscore [9] and DR [20] approaches.

## Methods

### Nonpher description

**Fig. 1:**
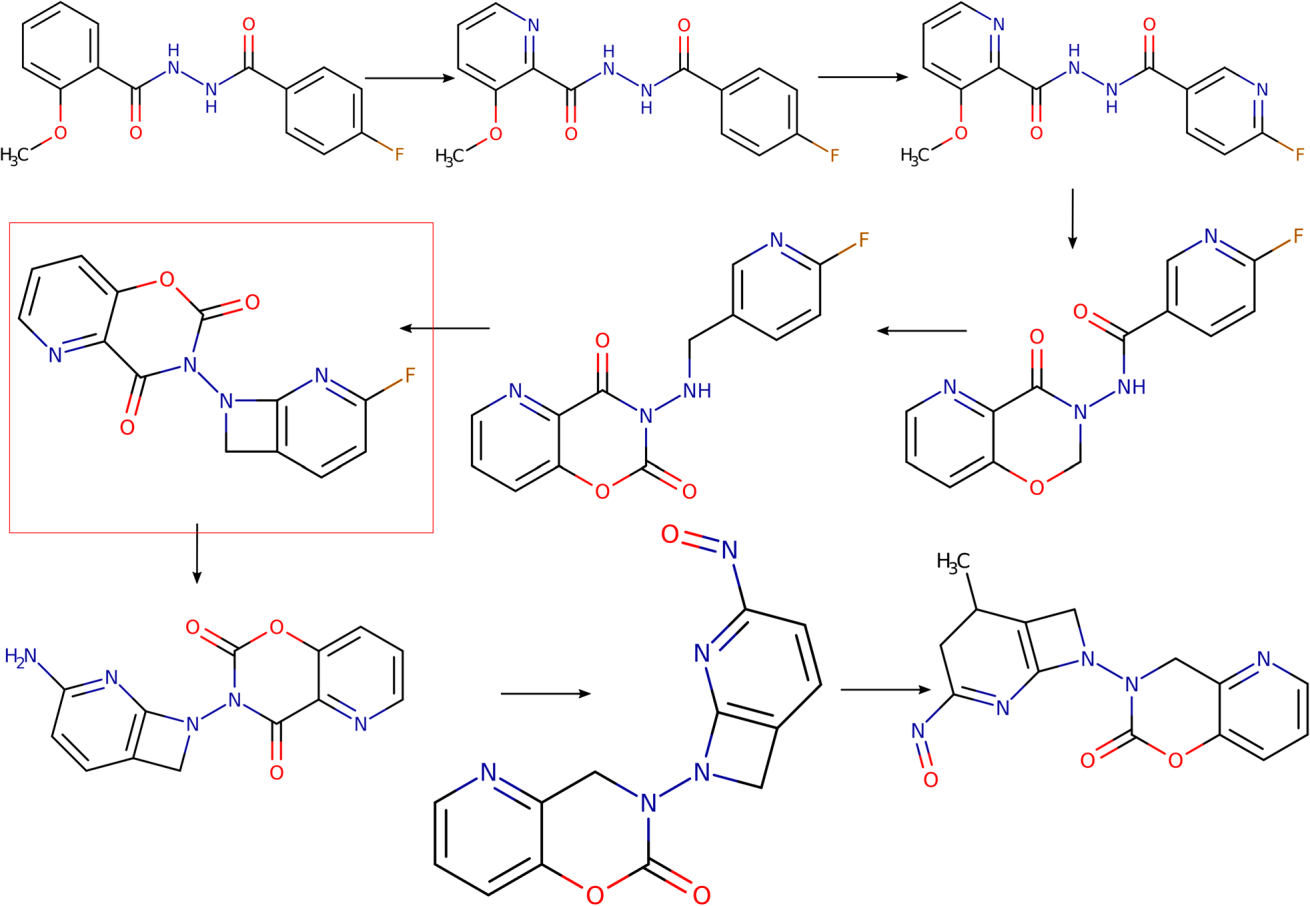
The example of the generation of a hard-to-synthesize compound. In molecular morphing, a path of molecules (morphs) that differ only by small structural perturbations is constructed. The compound in a *red rectangle* was identified as hard-to-synthesize, compounds beyond this point become overly complex

Nonpher is a methodology for the generation of hard-to-synthesize structures. The input to Nonpher is an existing structure, further referred to as a starting structure that gradually undergoes simple structural variations, such as the addition/removal/change of an atom or a bond. This process is called molecular morphing [23] and structures that form a morphing path are called morphs. In Nonpher, any structure can be used as a starting structure. A new morph is generated from the starting structure by a random choice of a structural variation (e.g., atom deletion) and by a random choice of a place in the starting structure where this variation is applied. The process is stochastic and generates, in a stepwise manner, linear paths from a starting structure. With the lengthening of a morphing path, morphs get more complex and after a certain number of steps they can be considered as synthetically unfeasible. However, if morphing is terminated too late, morphs get excessively complex (Fig. 1; Additional file 2: Figure S1). Thus, the number of morphing steps must be optimized so that structural variations that lead to a change in a synthetic accessibility are captured just after they appear. In Nonpher, the optimum number of morphing steps is obtained for each morphing path by monitoring morph complexity using the Bertz [15], Whitlock [16], BC [17], and SMCM [18] complexity indices as implemented in the RDKit cheminformatics toolkit [24], version Q1 2014.

### Construction of training set

To train a binary classifier, the training data set $S_{train}$ must consist of both positive (i.e., compounds that are relatively easy to synthesize) and negative (i.e., compounds that are hard to synthesize) examples. These subsets will be further denoted as $S_{train}^{ + }$ and $S_{train}^{ - }$, respectively. In the Nonpher approach, the $S_{train}^{ - }$ data set was generated by molecular morphing [23] as described in the previous section. The $S_{train}^{ + }$ data set was formed by compounds randomly chosen from the ZINC12 database [22]. ZINC12 contains over 30 million commercially available compounds and represents, after the exclusion of natural products, a reliable source of structures that can be prepared by current organic synthesis methods.

### Construction of test set

The performance of a binary classifier is assessed using a test set $S_{test}$ that consists of both positive ($S_{test}^{ + }$) and negative ($S_{test}^{ - }$) samples not used for model building [25]. To evaluate Nonpher performance, $S_{test}^{ - }$ compounds were obtained by the analysis of 296 published structures which ease of synthesis was assessed by experienced medicinal chemists. 12 compounds came from the SYLVIA paper [12], 100 structures from the RASA paper [11], 40 structures from the SAscore paper [9], and 144 molecules were randomly selected [26] from the KEGG DRUG database [27, 28]. Based both on original chemists’ scores, as well as on scores given by the SAscore [9], FA4 [26], SYLVIA [12] and RASA [11] methods, the final $S_{test}^{ - }$ data set of 40 hard-to-synthesize was assembled. A complementary $S_{test}^{ + }$ data set consists of 20 structures that were identified as easy-to-synthesize in the SAscore paper [9] enriched by 100 randomly selected ZINC12 structures. The test set is available in the SMILES format as Additional file 3.

### Construction of SAscore and DR sets

The quality of the Nonpher $S_{train}^{ - }$ library was compared with the $S_{train}^{ - }$ data sets constructed using the SAscore [9] and dense region (DR) [20] approaches. SAscore was calculated for the whole ZINC12 database (22,723,223 compounds) and 54,750 structures that exceeded the recommended threshold of 6 [9] formed the $S_{train}^{ - }$ data set. The same number of $S_{train}^{ + }$ structures were randomly selected from ZINC12 compounds with the SAscore lower than 4 (Additional file 4). This threshold was chosen to ensure that $S_{train}^{ + }$ and $S_{train}^{ - }$ sets contain compounds with markedly different complexities.

DR approach [20] is based on the assumption that synthetically feasible compound lies in rather dense part of chemical space (i.e., it has many structurally similar neighbors), while unfeasible compound occupies a sparse chemical space region. The $S_{train}^{ + }$ and $S_{train}^{ - }$ DR data sets were constructed from the Molecular Libraries Small Molecule Repository (MLSMR) downloaded from PubChem [21] on 15. 7. 2015. As recommended by the authors, $S_{train}^{ + }$ data set consists of MLSMR compounds with 20 or more nearest neighbors and $S_{train}^{ - }$ data set of MLSMR compounds with up to one neighbor [20]. To identify structurally similar neighbors, compounds were represented by 512-bit Morgan fingerprints with the radius of 2 (RDKit equivalent to widely adopted ECFP4 fingerprint [19]) and their similarity was assessed by the Tanimoto coefficient, threshold of which was set to 0.6. The $S_{train}^{ + }$ DR data set contains 113,176 structures and the $S_{train}^{ - }$ DR data set contains 50,345 structures (Additional file 5).

### Random forest classifier

The classification was performed by a random forest (RF), a method proven in various chemoinformatics applications [29–31]. In a random forest, the ensemble of decision trees using random subsets of features is generated from the bootstrapped sample of compounds. The advantage of a random forest is that no feature selection is required to achieve high classification accuracy and that predictions are rather robust to changes in model parameters. For classification purposes, all structures were encoded as 512-bit Morgan fingerprints with radius 2 and random forest classifier consisting of 100 trees implemented in Scikit-learn [32] was used.

The quality of proposed libraries of hard-to-synthesize structures was assessed by evaluating the performance of RF models that were built using individual $S_{train}^{ - }$ data sets augmented with corresponding $S_{train}^{ + }$ data sets. To assess RF model performance, overall classification accuracy *Acc*, a sensitivity *SN*, specificity *SP* and an area under a ROC curve *AUC* was calculated for a test set. A classification accuracy *Acc* gives the percentage of correctly classified samples regardless their class. Though *Acc* is a commonly used performance measure, it is less suitable for imbalanced data. A trivial classifier that assigns every data point into a majority class can still achieve a high accuracy. For imbalanced data, a classification accuracy can be calculated both for positive and negative classes independently. The percentage of a correctly predicted positive class is known as sensitivity (*SN*) and the percentage of a correctly predicted negative class is known as specificity (*SP*).

$$ {\text{Accuracy}}\, \left( {Acc} \right) = \frac{TP + TN}{TP + FN + FP + TN} $$

$$ {\text{Sensitivity}}\, \left( {\text{SN}} \right) = \frac{TP}{TP + FN} $$

$$ {\text{Specificity}}\, \left( {SP} \right) = \frac{TN}{TN + FP}. $$

These entities are defined using the following quantities: true positives (*TP*) are easy-to-synthesize structures predicted to be easy-to-synthesize, true negatives (*TN*) are hard-to-synthesize structures predicted to be hard-to-synthesize, false positives (*FP*) are hard-to-synthesize structures predicted to be easy-to-synthesize and false negatives (*FN*) are easy-to-synthesize structures predicted to be hard-to-synthesize.

*SN* and *SP* can be combined to create a two-dimensional receiver operating characteristic (ROC) curve that is the graphical representation of the trade-off between true positive rate (given as *SN*) and false positive rate (given as 1 − *SP*) over all possible thresholds. The area under the ROC curve (*AUC*) is the quantitative measure of the performance of a classifier and is equal to the probability that a classifier will rank a randomly chosen positive instance higher than a randomly chosen negative example. A random classifier has the *AUC* of 0.5, while the *AUC* for a perfect classifier is equal to 1.

## Results and discussion

To generate the Nonpher library of synthetically unfeasible structures, 500,000 compounds were randomly selected from the ZINC12 database [22]. These compounds served as starting structures for molecular morphing. For performance reasons, molecular morphing was terminated after 30 steps. $S_{train}^{ - }$ data set was formed by morphs complexity indices of which exceeded their thresholds within these 30 steps. In the present study, the following complexity indices were used: Bertz [15], Whitlock [16], BC [17], and SMCM [18] index. Their thresholds, used to distinguish between easy- and hard-to-synthesize structures, were determined by the analysis of the complexity distribution of 22,723,223 commercially available compounds from the ZINC12 database [22]. Because complexities are correlated with molecular weight (*MW*) [18], ZINC12 structures were binned by their *MW* into eleven intervals, each 50 Da wide. As expected, the medians of individual complexity indices increase with increasing *MW* (Additional file 2: Figure S2). For each bin and for each complexity index, its maximum number (i.e., all ZINC12 structures have lower complexity than a maximum), 999th permille (i.e., 99.9% ZINC12 structures have lower complexity then 999th permille) and 99th percentile (i.e., 99% ZINC12 structures have lower complexity than 99th percentile) were identified (Additional file 2: Table S1). The structures exceeding these limits are considered to be hard-to-synthesize. In addition to three possible complexity thresholds, also the number (1, 2, 3 or 4) of complexity indices that exceed these thresholds must be established. To select an optimal stop condition, twelve $S_{train}^{ - }$ data sets covering all possible combinations of up to four complexity indices (Bertz, Whitlock, BC, SMCM) exceeding each of three possible complexity thresholds (max, 999th permille and 99th percentile) were constructed. Each of the $S_{train}^{ - }$ data sets was augmented with the $S_{train}^{ + }$ data set consisting of corresponding starting structures. Thus, each $S_{train}$ data set contains the same number of positive and negative examples, but they differ in size. For each $S_{train}$ data set, a random forest was trained and its accuracy was assessed on the test set $S_{test}$. The highest classification accuracy (89.4%) and reasonably low and well balanced numbers of false negatives (9) and false positives (8) were obtained for structures that violate the 999th permille criterion for at least one complexity index (Additional file 2: Table S2). To make sure that the classification accuracy was not achieved by the fortunate selection of random structures, additional four $S_{train}^{ - }$ sets were generated from different randomly chosen ZINC12 structures (i.e., from different $S_{train}^{ + }$ sets). RF models trained on these data show comparable results (Additional file 2: Table S3) demonstrating the validity of suggested stopping criteria.

Similarly to the Nonpher database, ZINC and MLSMR $S_{train}^{ + }$ data sets were also sampled five times. In the case of the ZINC database, $S_{train}^{ - }$ data set is formed by 54,750 structures that exceed the SAscore threshold of 6. Five different $S_{train}^{ + }$ data sets were formed by random sampling of 54,750 structures from the ZINC12 database with the SAscore lower than 4. In DR method, 113,176 structures were identified as easy-to-synthesize and 50,345 structures as hard-to-synthesize. 50,345 hard-to-synthesize structures formed the $S_{train}^{ - }$ data set and five different $S_{train}^{ + }$ data sets of the same size were randomly sampled from 113,176 easy-to-synthesize DR structures. The average classifier performance for Nonpher, SAscore and DR methods is summarized in Table 1, performance measures of individual samples and their corresponding confusion matrices are available in Additional file 2: Tables S3–S5.

**Table 1:** Test set performances of random forest models trained on data generated by Nonpher, SAscore and DR approaches

Model	Acc (%)	SN (%)	SP (%)	AUC
Nonpher	89.6	93.8	77.0	0.94
SAscore	82.5	94.7	46.0	0.89
DR	46.0	30.8	91.5	0.60

An accuracy (Acc), sensitivity (SN), specificity (SP) and an area under a ROC curve (AUC) are calculated as average values from five different random samples of the $S_{train}$ data set

**Fig. 2:**
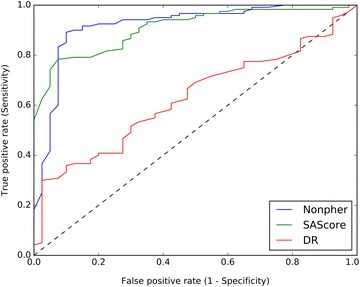
ROC curves (for a test set) of random forest models trained on data produced by Nonpher, SAscore and DR approaches

Of all three approaches, Nonpher produces hard-to-synthesize library with the highest classification accuracy of 89.6% and the best balance between a sensitivity and specificity (Table 1). The RF model trained on molecules selected according to their SAscore achieved the accuracy of 82.5% (AUC 0.89) and training data created by the DR method lead only to the accuracy of 46.0% (AUC 0.60). While *SN* and *SP* are reasonably balanced for the Nonpher data set, SAscore data yields a model with low *SP* and DR data with low *SN*. Thus, both SAscore and DR methods are deficient in obtaining too many false predictions. While SAscore model tends to predict more compounds as easy-to-synthesize, DR model labels a majority of compounds as hard-to-synthesize. Worse performance of DR model is also apparent from its lower AUC (Table 1) as well as from its ROC curve (Fig. 2). The inspection of ROC curves of Nonpher and SAscore models reveals that although SAscore model produces better ROC values for higher thresholds, Nonpher model is better at distinguishing hard-to-synthesize from easy-to-synthesize structures.

## Conclusions

Nonpher, our approach for the in silico generation of hard-to-synthesize structures, provides an important addition to existing tools for a computer-aided molecular design. In Nonpher, molecular morphing [23], that systematically alters a given structure by small structural changes (e.g., add or remove atom or bond), is used to construct hard-to-synthesize structures. The length of molecular morphing was optimized so that generated structures are hard-to-synthesize, although not overly complex. The quality of the Nonpher library was assessed by building a random forest (RF) classifier using a training set consisting of the Nonpher library augmented with synthetically accessible compounds randomly selected from the ZINC12 database [22]. The quality of RF model was verified by predicting the synthetic accessibility of 40 compounds that were carefully collected from the literature and were considered to be hard-to-synthesize by experienced medicinal and organic chemists [9, 11, 12, 26]. Nonpher was compared with SAscore [9] and DR [20] approaches, two alternative methods for the construction of hard-to-synthesize compounds. Nonpher yielded data of higher quality than both SAscore and DR methods, as demonstrated by a lower amount of false predictions and by better balance between sensitivity and specificity of Nonpher model. The Nonpher library of hard-to-synthesize compounds contains 1,706,950 structures and is available for download (Additional file 1). Similarly, test set consisting of 40 manually curated hard-to-synthesize compounds augmented with 120 easy-to-synthesize structures is also available (Additional file 3). By providing both training and test data sets we believe that our method will further boost research in the automatic prediction of molecular synthetic feasibility.

## Additional files

**Additional file 1.** Nonpher training set. It contains SMILES of 1,706,950 hard-to-synthesize structures and the same amount of easy-to-synthesize structures.

**Additional file 2.** The supporting information contains details on molecular complexities and on molecular morphing threshold optimization.

**Additional file 3.** Test set. It contains SMILES of 40 structures considered as hard-to-synthesize by medicinal chemists and 120 easy-to-synthesize structures.

**Additional file 4.** SAscore training set. It contains SMILES of 54,750 structures considered to be hard-to-synthesize by the SAscore criterion (i.e., with SAscore higher than 6) and the same amount of easy-to-synthesize structures.

**Additional file 5.** DR training set. It contains SMILES of 50,345 hard-to-synthesize structures (i.e., they are from a sparse region) and of 113,176 easy-to-synthesize structures (i.e., they are from a dense region).

## Abbreviations

RF: random forest
TP: true positives
TN: true negatives
FP: false positives
FN: false negatives
Acc: accuracy
SN: sensitivity
SP: specificity
ROC: receiver operating characteristic
AUC: area under receiver operating characteristic
DR: dense region
BC: Barone and Chanon complexity index
SMCM: synthetic and molecular complexity metric
MLSMR: Molecular Libraries Small Molecule Repository
MW: molecular weight

## References

[1] Klebe G (2006). Virtual ligand screening: strategies, perspectives and limitations. Drug Discov Today.

[2] Shoichet BK (2004). Virtual screening of chemical libraries. Nature.

[3] Hartenfeller M, Schneider G, Bajorath J (2011). De Novo Drug Design. Chemoinformatics and computational chemical biology.

[4] Bonnet P (2012). Is chemical synthetic accessibility computationally predictable for drug and lead-like molecules? A comparative assessment between medicinal and computational chemists. Eur J Med Chem.

[5] Lajiness MS, Maggiora GM, Shanmugasundaram V (2004). Assessment of the consistency of medicinal chemists in reviewing sets of compounds. J Med Chem.

[6] Takaoka Y, Endo Y, Yamanobe S, Kakinuma H, Okubo T, Shimazaki Y, Ota T, Sumiya S, Yoshikawa K (2003). Development of a method for evaluating drug-likeness and ease of synthesis using a data set in which compounds are assigned scores based on chemists’ intuition. J Chem Inf Comput Sci.

[7] Kutchukian PS, Vasilyeva NY, Xu J, Lindvall MK, Dillon MP, Glick M, Coley JD, Brooijmans N (2012). Inside the mind of a medicinal chemist: the role of human bias in compound prioritization during drug discovery. PLoS ONE.

[8] Baber JC, Feher M (2004). Predicting synthetic accessibility: application in drug discovery and development. Mini Rev Med Chem.

[9] Ertl P, Schuffenhauer A (2009). Estimation of synthetic accessibility score of drug-like molecules based on molecular complexity and fragment contributions. J Cheminform.

[10] Ihlenfeldt W-D, Gasteiger J (1996). Computer-assisted planning of organic syntheses: the second generation of programs. Angew Chem Int Ed Engl.

[11] Huang Q, Li L-L, Yang S-Y (2011). RASA: a rapid retrosynthesis-based scoring method for the assessment of synthetic accessibility of drug-like molecules. J Chem Inf Model.

[12] Boda K, Seidel T, Gasteiger J (2007). Structure and reaction based evaluation of synthetic accessibility. J Comput Aided Mol Des.

[13] Gillet VJ, Myatt G, Zsoldos Z, Johnson AP (1995). SPROUT, HIPPO and CAESA: Tools for de novo structure generation and estimation of synthetic accessibility. Perspect Drug Discov Des.

[14] Selzer P, Roth H-J, Ertl P, Schuffenhauer A (2005). Complex molecules: do they add value?. Curr Opin Chem Biol.

[15] Bertz SH (1981). The first general index of molecular complexity. J Am Chem Soc.

[16] Whitlock HW (1998). On the structure of total synthesis of complex natural products. J Org Chem.

[17] Barone R, Chanon M (2001). A new and simple approach to chemical complexity. Application to the synthesis of natural products. J Chem Inf Comput Sci.

[18] Allu TK, Oprea TI (2005). Rapid evaluation of synthetic and molecular complexity for in silico chemistry. J Chem Inf Model.

[19] Rogers D, Hahn M (2010). Extended-connectivity fingerprints. J Chem Inf Model.

[20] Podolyan Y, Walters MA, Karypis G (2010). Assessing synthetic accessibility of chemical compounds using machine learning methods. J Chem Inf Model.

[21] Kim S, Thiessen PA, Bolton EE, Chen J, Fu G, Gindulyte A, Han L, He J, He S, Shoemaker BA (2016). PubChem substance and compound databases. Nucleic Acids Res.

[22] Irwin JJ, Sterling T, Mysinger MM, Bolstad ES, Coleman RG (2012). ZINC: a free tool to discover chemistry for biology. J Chem Inf Model.

[23] Hoksza D, Skoda P, Vorsilak M, Svozil D (2014). Molpher: a software framework for systematic chemical space exploration. J Cheminform.

[24] RDKit: Open-source cheminformatics. http://www.rdkit.org

[25] Bishop C (2007). Pattern recognition and machine learning.

[26] Fukunishi Y, Kurosawa T, Mikami Y, Nakamura H (2014). Prediction of synthetic accessibility based on commercially available compound databases. J Chem Inf Model.

[27] Kanehisa M, Goto S, Sato Y, Furumichi M, Tanabe M (2012). KEGG for integration and interpretation of large-scale molecular data sets. Nucleic Acids Res.

[28] Kanehisa M, Sato Y, Kawashima M, Furumichi M, Tanabe M (2016). KEGG as a reference resource for gene and protein annotation. Nucleic Acids Res.

[29] Svetnik V, Liaw A, Tong C, Culberson JC, Sheridan RP, Feuston BP (2003). Random Forest: a Classification and Regression Tool for Compound Classification and QSAR Modeling. J Chem Inf Comput Sci.

[30] Palmer DS, O’Boyle NM, Glen RC, Mitchell JBO (2007). Random Forest Models To Predict Aqueous Solubility. J Chem Inf Model.

[31] Bruce CL, Melville JL, Pickett SD, Hirst JD (2007). Contemporary QSAR Classifiers Compared. J Chem Inf Model.

[32] Pedregosa F, Varoquaux G, Gramfort A, Michel V, Thirion B, Grisel O, Blondel M, Prettenhofer P, Weiss R, Dubourg V (2011). Scikit-learn: machine Learning in Python. Journal of Machine Learning Research.

