# Supplementary material for: Nonpher: computational method for design of hard-to-synthesize structures
Source: J Cheminform. 2017 Mar 20;9:20. doi: 10.1186/s13321-017-0206-2 (PMC5359269; doi:10.1186/s13321-017-0206-2)
Supplement: Supplementary file 2 — Additional file 2. The supporting information contains details on molecular complexities and on molecular morphing threshold optimization. [file 13321_2017_206_MOESM2_ESM.docx]

**Additional file**

**Nonpher: computational method for design of hard-to-synthesize structures**

Milan Voršilák^1^, Daniel Svozil ^1, 2,*^

^1^ CZ-OPENSCREEN:National Infrastructure for Chemical Biology, Laboratory of Informatics and Chemistry, Faculty of Chemical Technology, University of Chemistry and Technology Prague, Prague, Czech Republic

^2^ CZ-OPENSCREEN: National Infrastructure for Chemical Biology, Institute of Molecular Genetics, AS CR v.v.i., Prague, Czech Republic

^*^Corresponding author

Email addresses:

MV: [Milan.Vorsilak@vscht.cz](mailto:Milan.Vorsilak@vscht.cz)

DS: [Daniel.Svozil@vscht.cz](mailto:Daniel.Svozil@vscht.cz)

**Figure S1.** The time progression of complexity indices. Molecular morphing of 320,000 structures was terminated after 30^th^ step. At each step, a box plot (red line inside a box shows a median, whiskers lie 1.5x*IQR* (interquartile range) below/above first/third quartile, respectively) depicts the distribution of the complexity index of 320,000 compounds. The complexity of morphs steadily increases with time.

Bertz complexity index


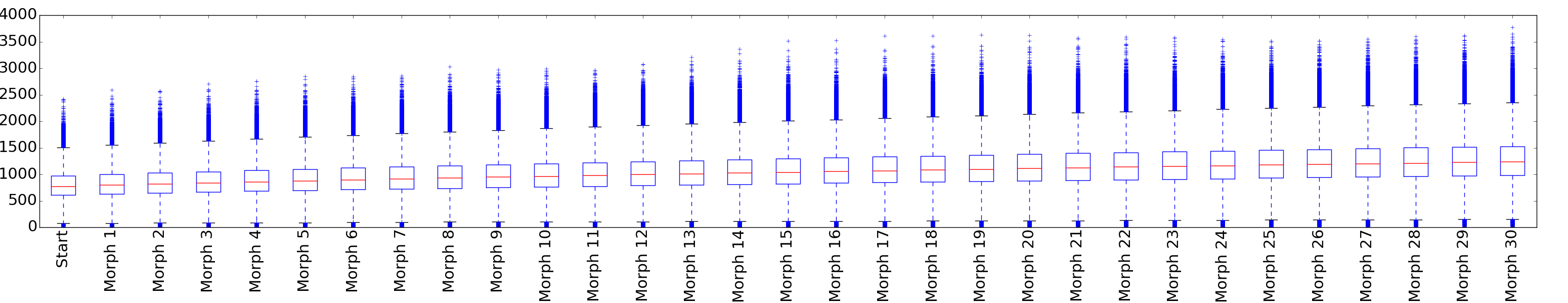


Barone complexity index


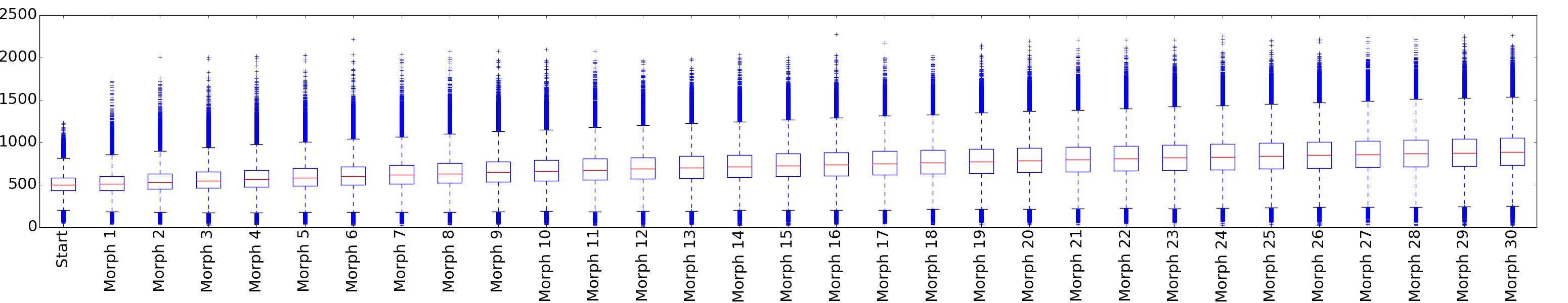


SMCM complexity index


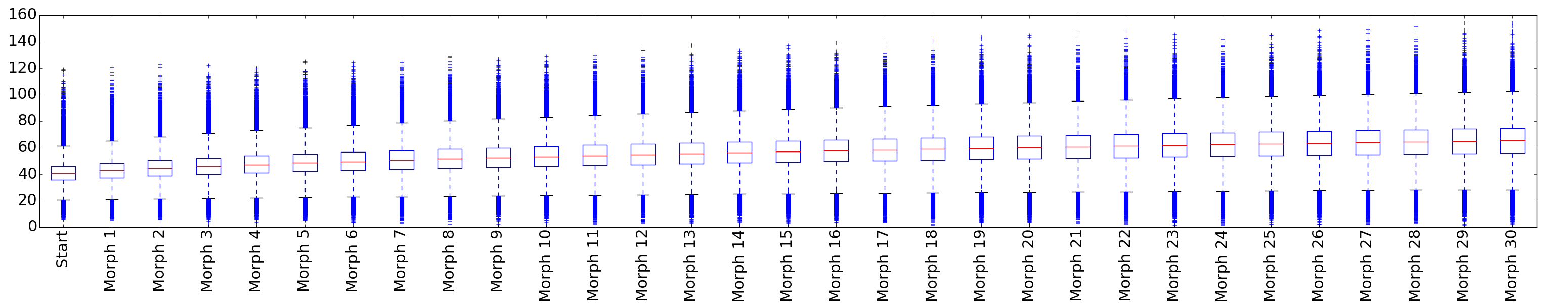


Whitlock complexity index


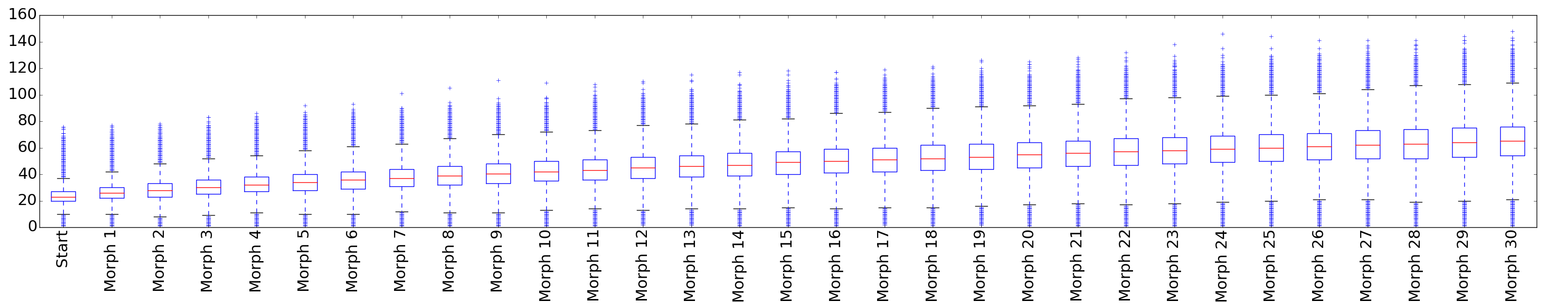


**Figure S2.** The dependency of complexity indices on a molecular weight. All 22,723,223 ZINC structures were divided into 11 bins, each 50 Da wide. The number of structures in individual bins is shown in parentheses: <150 Da (53 923), 150-200 Da (306 215), 200-250 Da (1 420 340), 250-300 Da (4 006 064), 300-350 Da (6 602 461), 350-400 Da (5 692 467), 400-450 Da (2 602 706), 450-500 Da (1 624 209), 500-550 Da (374 974), 550-600 Da (28 116), >600 Da (11 748). Each box plot shows a median (red line), whiskers lie 1.5x*IQR* (interquartile range) below/above first/third quartile, respectively.

Bertz complexity


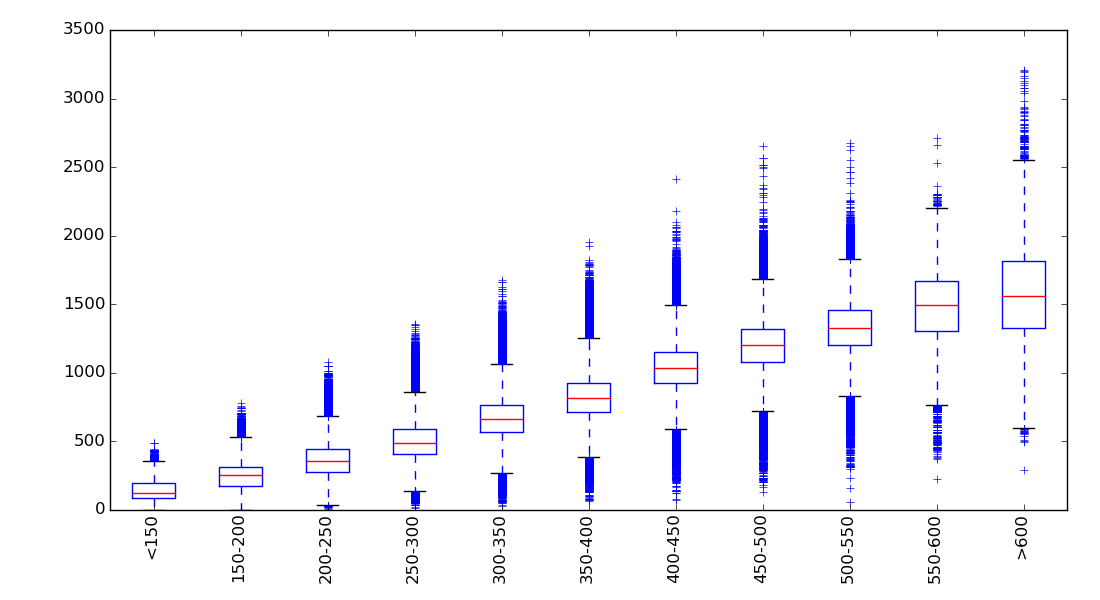
Barone complexity
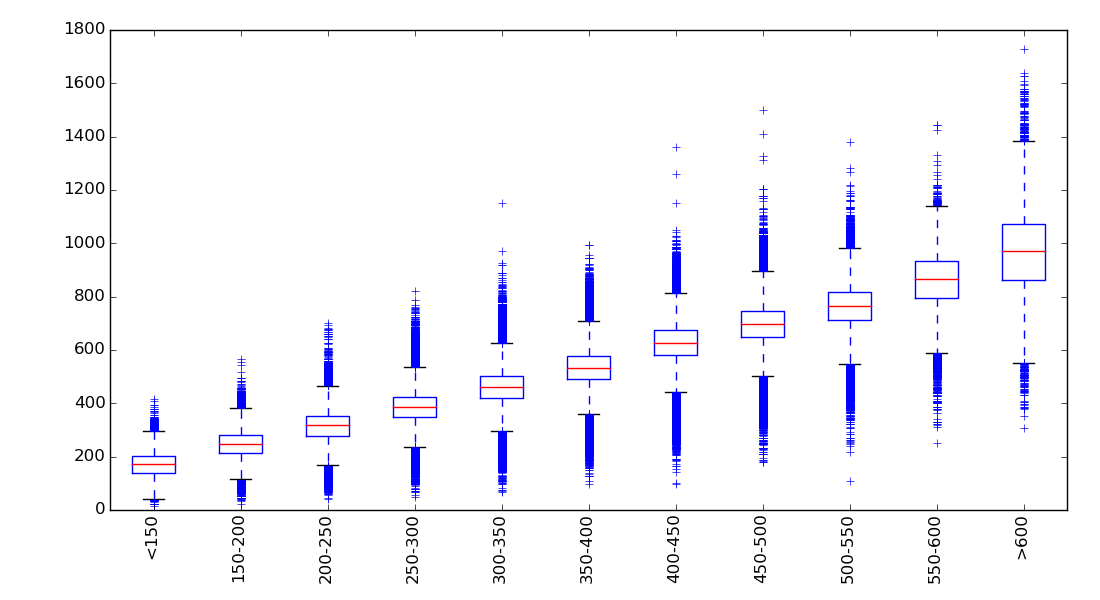


SMCM complexity


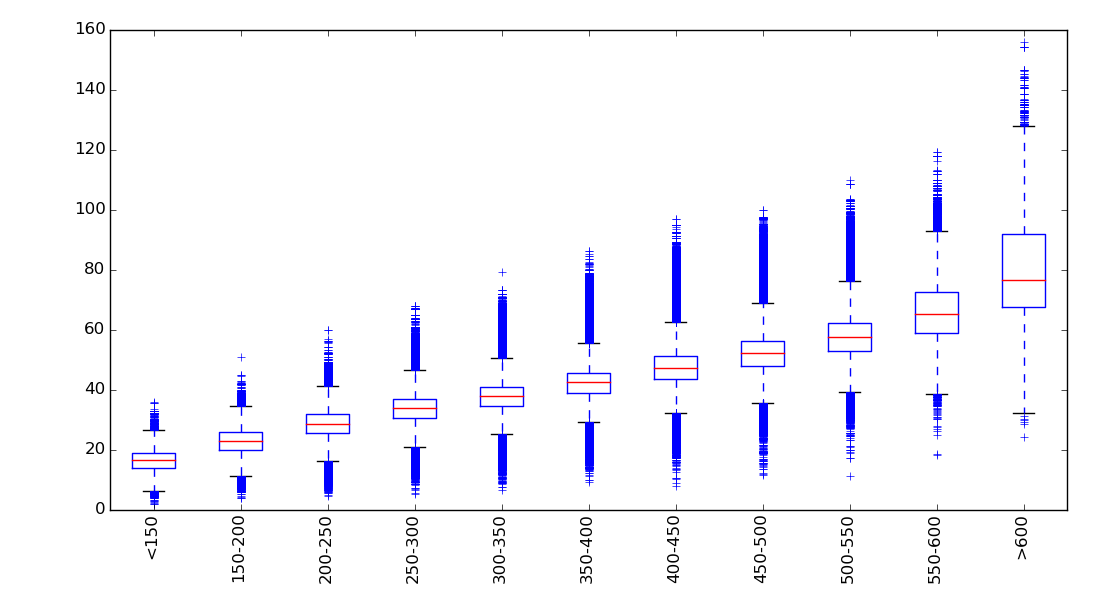


Whitlock complexity


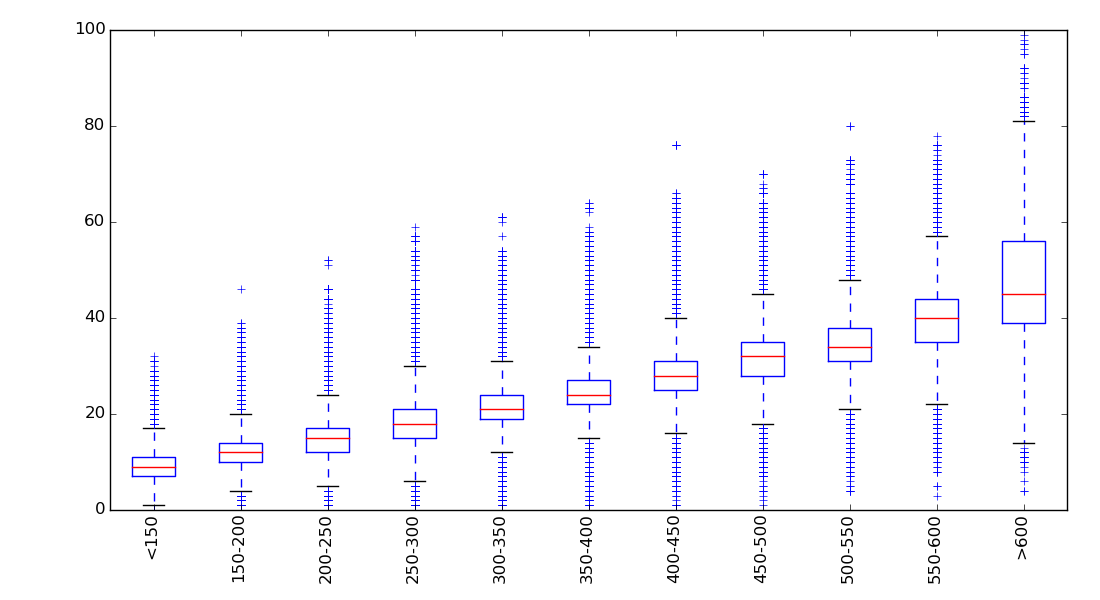


**Table S1.** Boundary values of ZINC compound complexity indices within individual *MW* bins. 99^th^ percentile means that 99% ZINC structures have a complexity lower than a given value, 999^th^ permille is the same for 99.9% structures, and maximum is the maximal value of a complexity index in the ZINC database. For example, 99% ZINC compounds with *MW* between 150 Da and 200 Da have Bertz index lower than 394.

99^th^ percentile

| Complexity index | bin (Da) | | | | | | | | | | |
| --- | --- | --- | --- | --- | --- | --- | --- | --- | --- | --- | --- |
|  | <150 | <200 | <250 | <300 | <350 | <400 | <450 | <500 | <550 | <600 | >600 |
| Bertz | 257 | 394 | 525 | 679 | 864 | 1036 | 1261 | 1435 | 1573 | 1826 | 2051 |
| Barone | 234 | 309 | 384 | 462 | 540 | 619 | 717 | 794 | 866 | 1002 | 1174 |
| Whitlock | 14 | 17 | 20 | 23 | 26 | 30 | 34 | 38 | 41 | 51 | 67 |
| SMCM | 21.7 | 28.8 | 35.0 | 40.2 | 44.0 | 48.8 | 54.9 | 60.5 | 67.0 | 81.7 | 107.6 |

999^th^ permille

| Complexity index | bin (Da) | | | | | | | | | | |
| --- | --- | --- | --- | --- | --- | --- | --- | --- | --- | --- | --- |
|  | <150 | <200 | <250 | <300 | <350 | <400 | <450 | <500 | <550 | <600 | >600 |
| Bertz | 390 | 520 | 673 | 845 | 1043 | 1230 | 1456 | 1644 | 1784 | 2052 | 2540 |
| Barone | 281 | 360 | 438 | 522 | 607 | 695 | 795 | 884 | 968 | 1085 | 1408 |
| Whitlock | 19 | 22 | 25 | 28 | 32 | 36 | 42 | 47 | 51 | 64 | 81 |
| SMCM | 21.7 | 33.7 | 40.1 | 46.2 | 49.5 | 55.2 | 63.5 | 70.0 | 81.3 | 100.7 | 128.9 |

Maximum

| Complexity index | bin (Da) | | | | | | | | | | |
| --- | --- | --- | --- | --- | --- | --- | --- | --- | --- | --- | --- |
|  | <150 | <200 | <250 | <300 | <350 | <400 | <450 | <500 | <550 | <600 | >600 |
| Bertz | 487 | 782 | 1078 | 1358 | 1675 | 1957 | 2417 | 2654 | 2675 | 2714 | 3210 |
| Barone | 418 | 566 | 702 | 822 | 1152 | 995 | 2250 | 1500 | 3636 | 1445 | 4668 |
| Whitlock | 32 | 46 | 52 | 59 | 61 | 64 | 76 | 70 | 80 | 78 | 100 |
| SMCM | 36.0 | 50.8 | 60.1 | 68.1 | 79.4 | 68.3 | 96.9 | 100.1 | 109.8 | 119.2 | 156.1 |

**Table S2.** Test set prediction results of random forest models trained on Nonpher data obtained with different settings. Different complexity thresholds (99^th^ percentile, 999^th^ permille and a maximum) and the different number of violations (between 1 and 4) were tested. Molecular morphing is stopped when at least one complexity index (no matter which one) exceeds its 999^th^ permille value (for a given *MW* bin). An accuracy, a sensitivity and specificity for this setting are shown in red. n – the size of the whole training set, Acc – an accuracy, SN – a sensitivity, SP – a specificity, AUC – the area under the ROC curve, TP – true positives, FN – false negatives, FP – false positives, TN – true negatives

| Complexity threshold | # of violations | n | Acc | SN | SP | AUC | TP | FN | FP | TN |
| --- | --- | --- | --- | --- | --- | --- | --- | --- | --- | --- |
| 99% | 1 | 541,276 | 85.0 | 83.3 | 90.0 | 0.92 | 100 | 20 | 4 | 36 |
|  | 2 | 591,194 | 85.6 | 86.7 | 82.5 | 0.93 | 104 | 16 | 7 | 33 |
|  | 3 | 530,754 | 86.9 | 92.5 | 70.0 | 0.93 | 111 | 9 | 12 | 28 |
|  | 4 | 250,060 | 85.6 | 95.0 | 57.5 | 0.93 | 114 | 6 | 17 | 23 |
| 99.9% | 1 | 638,178 | **89.4** | **92.5** | **80.0** | 0.92 | 111 | 9 | 8 | 32 |
|  | 2 | 548,530 | 86.3 | 95.0 | 60.0 | 0.92 | 114 | 6 | 16 | 24 |
|  | 3 | 326,106 | 85.3 | 95.0 | 57.5 | 0.92 | 114 | 6 | 17 | 23 |
|  | 4 | 80,660 | 84.4 | 98.3 | 42.5 | 0.92 | 118 | 2 | 23 | 17 |
| Maximum | 1 | 207,664 | 79.4 | 97.5 | 25.0 | 0.90 | 117 | 3 | 30 | 10 |
|  | 2 | 59,152 | 80.0 | 99.2 | 22.5 | 0.89 | 119 | 1 | 31 | 9 |
|  | 3 | 2,152 | 76.3 | 100.0 | 5.0 | 0.89 | 120 | 0 | 38 | 2 |
|  | 4 | 34 | 75.6 | 100.0 | 2.5 | 0.89 | 120 | 0 | 39 | 1 |

**Table S3.** Test set prediction results of random forest models trained on Nonpher data obtained with 999^th^ permille/1 violation setting. Each of five data sets was constructed from 500,000 randomly chosen ZINC12 structures. n – the size of the whole training set, Acc – an accuracy, SN – a sensitivity, SP – a specificity, AUC – the area under the ROC curve, TP – true positives, FN – false negatives, FP – false positives, TN – true negatives

| Data set # | n | Acc | SN | SP | AUC | TP | FN | FP | TN |
| --- | --- | --- | --- | --- | --- | --- | --- | --- | --- |
| 1 | 696,452 | 90.0 | 94.2 | 77.5 | 0.94 | 113 | 7 | 9 | 31 |
| 2 | 692,564 | 89.4 | 95.0 | 72.5 | 0.94 | 114 | 6 | 11 | 29 |
| 3 | 693,194 | 90.6 | 95.0 | 77.5 | 0.94 | 114 | 6 | 9 | 31 |
| 4 | 693,512 | 91.9 | 95.8 | 80.0 | 0.94 | 115 | 5 | 8 | 32 |
| 5 | 638,178 | 89.4 | 92.5 | 80.0 | 0.92 | 111 | 9 | 8 | 32 |
| average | - | 89.6 | 93.8 | 77.0 | 0.94 | - | - | - | - |

**Table S4.** Test set prediction results of random forest models trained on SAscore data obtained from the ZINC12 database. $S_{train}^{-}$ data set is formed by 54,750 structures that exceed the SAscore threshold of 6. Five different $S_{train}^{+}$ data sets were formed by random sampling of 54,750 structures from the ZINC12 database with the SAscore lower than 4. n – the size of the whole training set, Acc – an accuracy, SN – a sensitivity, SP – a specificity, AUC – the area under the ROC curve, TP – true positives, FN – false negatives, FP – false positives, TN – true negatives

| Data set # | n | Acc | SN | SP | AUC | TP | FN | FP | TN |
| --- | --- | --- | --- | --- | --- | --- | --- | --- | --- |
| 1 | 109,500 | 81.3 | 93.3 | 45.0 | 0.89 | 112 | 8 | 22 | 18 |
| 2 | 109,500 | 83.8 | 95.0 | 50.0 | 0.89 | 114 | 6 | 20 | 20 |
| 3 | 109,500 | 83.1 | 95.0 | 47.5 | 0.90 | 114 | 6 | 21 | 19 |
| 4 | 109,500 | 83.1 | 95.8 | 45.0 | 0.90 | 115 | 5 | 22 | 18 |
| 5 | 109,500 | 81.3 | 94.2 | 42.5 | 0.88 | 113 | 7 | 23 | 17 |
| average |  | 82.5 | 94.7 | 46.0 | 0.89 | - | - | - | - |

**Table S5.** Test set prediction results of random forest models trained on DR data obtained from the MLSMR database. 113,176 MLSMR structures were identified as easy-to-synthesize and 50,345 MLSMR structures as hard-to-synthesize. 50,345 hard-to-synthesize structures formed the $S_{train}^{-}$ data set and five different $S_{train}^{+}$ data sets of the same size were randomly sampled from 113,176 easy-to-synthesize DR structures. n – the size of the whole training set, Acc – an accuracy, SN – a sensitivity, SP – a specificity, AUC – the area under the ROC curve, TP – true positives, FN – false negatives, FP – false positives, TN – true negatives

| Data set # | n | Acc | SN | SP | AUC | TP | FN | FP | TN |
| --- | --- | --- | --- | --- | --- | --- | --- | --- | --- |
| 1 | 100,690 | 44.4 | 30.0 | 87.5 | 0.60 | 36 | 84 | 5 | 35 |
| 2 | 100,690 | 46.3 | 31.7 | 90.0 | 0.60 | 38 | 82 | 4 | 36 |
| 3 | 100,690 | 46.9 | 31.7 | 92.5 | 0.61 | 38 | 82 | 3 | 37 |
| 4 | 100,690 | 46.3 | 29.2 | 97.5 | 0.59 | 35 | 85 | 1 | 39 |
| 5 | 100,690 | 46.3 | 31.7 | 90.0 | 0.60 | 38 | 82 | 4 | 36 |
| average | - | 46.0 | 30.8 | 91.5 | 0.60 | - | - | - | - |
